# Predictors of Suicidal Ideation and Preparatory Behaviors in patients With Bipolar Disorder: The Contribution of Chronobiological alterations and Its Association With Hopelessness

**DOI:** 10.1192/j.eurpsy.2023.266

**Published:** 2023-07-19

**Authors:** L. Palagini, A. Gemignani, L. Grassi, P. A. Geoffroy

**Affiliations:** 1 University of Ferrara, ferrara; 2 Univrsity of Pisa, Pisa, Italy; 3 University of Paris, Paris, France

## Abstract

**Introduction:**

Bpolar disorder (BD) is a severe and chronic psychiatric disorder it is the sixth leading cause of disability among all illnesses worldwide. With regard to causes of premature mortality, patients with BD are at very high risk of suicide. Whereas risk factors for suicidal behaviors are multiple and complex, hopelessness appears to be a major independent risk factor for suicidality in BD. Compelling evidence has also demonstrated that BD is frequently associated with circadian rhythms alteration, contributing to its vulnerability, pathogenesis, and manifestations. in particular in the desynchronization of sleep and social life, has been associated with the severity of BD. Indeed hopelessness has never been studid in relation to circadian rhythms in BD

**Objectives:**

To examine the role of chronobiological rhythm alterations in suicidal ideation and behaviors and its relation with hopelessness.

**Methods:**

One hundred twenty-seven patients (77 females, mean age of 47.4 ± 12.5 years) with a major depressive episode and bipolar disorder (BD) type I or II (according to Structured Clinical Interview for DSM-5 assessment) were recruited in 2019 and assessed for depressive and manic symptoms (Beck Depression Inventory-II, Young Mania Rating Scale) and with the Biological Rhythms Interview of Assessment in Neuropsychiatry, Beck Hopelessness Scale, and Scale for Suicide Ideation. Univariate regression and mediation analyses were performed.

**Results:**

Forty-one patients (32.3%) showed clinically significant suicidal ideation and were more frequently affected by BD type I (P = .029) with mixed features (P = .022). Compared to nonsuicidal individuals, they had significantly more depressive symptoms (P = .019), higher emotional component of hopelessness (P = .037), and higher dysrhythmicity of sleep (P = .009), activities (P = .048), and social life (P = .019). Passive and active suicidal ideation and suicidal plans were best predicted by dysrhythmicity of sleep and social life. Dysrhythmicity of sleep and social life mediated the direct effect of depressive symptoms on passive and active suicidal ideation and also of active ideation on suicidal plans. The emotional component of hopelessness was related to dysrhythmicity of social life and mediated its effect on suicidal plans (P = .010).

**Conclusions:**

Chronobiological alterations directly contributed to passive and active suicidal ideation and to suicidal preparation, with a key role of circadian rhythm alteration of sleep, activities, and social life. Chronobiological alterations also impacted the emotional component of hopelessness, hence indirectly contributing to suicidal ideations and plans. These findings call for the systematic screening of these dysrhythmicity dimensions when considering suicidal risk in individuals with BD.

**Disclosure of Interest:**

None Declared

